# Self-Reported Dietary Attentiveness to Fruit and Vegetable Intake and Actigraphy-Measured Sleep Efficiency in Middle-Aged and Older Women: A Cross-Sectional Study

**DOI:** 10.3390/nu18122027

**Published:** 2026-06-22

**Authors:** Chun-Hao Chen, Hsiao-Han Tang, I-Ju Lai, Yi-Chen Lee, Szu-Yu Hou, Ching-Ju Chiu

**Affiliations:** 1 Institute of Gerontology, College of Medicine, National Cheng Kung University, Tainan 701401, Taiwan; 2 Nutritec Care Nutrition Center, Taipei 111054, Taiwan; 3 Department of Nutrition, College of Medicine, I-Shou University, Kaohsiung 824005, Taiwan; 4 Department of Nutrition Therapy, E-Da Hospital, Kaohsiung 824005, Taiwan; 5 Department of Nutrition Therapy, E-Da Cancer Hospital, Kaohsiung 824005, Taiwan; 6 Institute of Health Policy and Management, College of Public Health, National Taiwan University, Taipei 100025, Taiwan

**Keywords:** fruit and vegetable intake, sleep efficiency, actigraphy, middle-aged and older women, dietary behavior

## Abstract

**Background/Objectives**: Diet and sleep are both important modifiable factors in healthy aging, yet little is known about whether attention to healthy eating behaviors is associated with objectively measured sleep. This study examined the association between self-reported attention to fruit and vegetable intake and actigraphy-measured sleep efficiency among women aged 45 years and older. **Methods**: This cross-sectional study included 143 women aged 45 years and older recruited from community centers. Participants wore a wrist-worn actigraphy device continuously for 7 days and completed daily sleep logs. Attention to fruit and vegetable intake was assessed using a single-item, four-category self-report measure. Hierarchical multiple linear regression was used to examine its independent association with sleep efficiency after adjustment for sociodemographic, health-related, and psychological covariates. **Results**: The mean age of the participants was 56.34 ± 7.67 years, and the mean sleep efficiency was 82.69 ± 8.60%. In the fully adjusted model, participants who reported “often” paying attention to fruit and vegetable intake had significantly higher sleep efficiency than those who reported doing so “almost every day” (β = 0.24, *p* = 0.013). Older age was independently associated with lower sleep efficiency (β = −0.31, *p* = 0.001). **Conclusions**: In this exploratory cross-sectional study, a single-item measure of self-reported attentiveness to fruit and vegetable intake showed a category-specific association with actigraphy-measured sleep efficiency. Longitudinal studies using more detailed dietary and behavioral measures are needed to clarify the direction and meaning of this association.

## 1. Introduction

Aging is accompanied by substantial physiological and behavioral changes that shape women’s long-term health trajectories. Women generally live longer than men and therefore spend a greater proportion of their lives in midlife and older adulthood, a period during which sleep and circadian regulation become increasingly relevant to healthy aging. During this stage, age-related alterations in the circadian system become more pronounced. Circadian rhythm plays a critical role in regulating core physiological functions, and persistent circadian disruption has been linked to adverse health outcomes [1]. With advancing age, circadian systems undergo significant modifications affecting behavioral rhythms, thermoregulation, and hormonal secretion, which are directly associated with decreased quality of life and increased disease risk [2,3]. Sleep disturbance has also been associated with cellular aging, metabolic dysregulation, and elevated risks of cardiovascular disease, neurodegenerative disorders, sleep-disordered breathing, and frailty [4,5,6,7]. Given the central role of sleep and circadian regulation in healthy aging, identifying modifiable lifestyle factors associated with sleep efficiency is of considerable importance, particularly among women aged 45 years and older.

Dietary behavior represents one of the most modifiable lifestyle factors influencing health in later life. Diet is closely associated with overall health status and quality of life among older adults [8,9]. Emerging evidence suggests that overall dietary quality and specific dietary patterns are related to sleep outcomes. Higher adherence to healthy dietary patterns, such as the DASH diet or other high-quality diet indices, has been associated with better sleep quality [10,11,12]. In addition, fruit and vegetable intake has received particular attention. Short sleep duration has been associated with a lower likelihood of consuming fruits and vegetables [13,14], and occasional consumption of fruits and vegetables has been linked to poorer sleep quality compared with daily intake [15]. These findings suggest that dietary behaviors—especially fruit and vegetable consumption—may play a role in sleep health. Recent evidence from a systematic review and meta-analysis further supports an association between dietary patterns and insomnia-related outcomes, thereby providing a broader context for diet–sleep links in midlife and older adulthood [16].

Beyond dietary composition and intake quantity, emerging work in chrono-nutrition highlights the bidirectional relationship between eating behaviors and circadian regulation. Irregular eating patterns may disrupt endogenous circadian systems [17,18], whereas circadian disruption itself contributes to metabolic abnormalities and increased risks of hypertension, dyslipidemia, impaired glucose regulation, and abdominal obesity [19,20]. However, most existing studies focus primarily on quantified nutrient intake, dietary indices, or meal timing. Relatively limited attention has been paid to the behavioral dimension of healthy eating—namely, whether individuals actively attend to and monitor their dietary practices in daily life. This distinction is important because attention to fruit and vegetable intake may not necessarily indicate higher actual intake or nutritional adequacy; instead, it may reflect a broader orientation toward health-related self-monitoring and daily behavioral organization. In the present study, self-reported attentiveness to daily fruit and vegetable intake was not conceptualized as a direct measure of actual intake quantity, dietary adequacy, or adherence to nutritional recommendations. Rather, it was treated as a brief exploratory behavioral marker reflecting participants’ perceived attention to health-oriented eating in everyday life. From a theoretical perspective, this construct may overlap with health consciousness, self-regulation, and lifestyle organization. Health consciousness refers to the extent to which individuals are aware of and concerned about health-related behaviors, whereas self-regulation involves setting goals, monitoring behavior, and adjusting actions when behavior deviates from intentions [21]. In this context, paying attention to fruit and vegetable intake may serve as a simple indicator of an individual’s tendency to monitor health-relevant routines rather than as a validated dietary assessment tool. Individuals who actively monitor dietary practices may also maintain more structured daily routines and better behavioral regularity, which could plausibly support more stable sleep–wake behaviors and better sleep outcomes [22]. In addition, health-related behaviors often cluster together; thus, attentional engagement in dietary practices may co-occur with other routine-supportive behaviors that collectively reinforce sleep health [23]. Accordingly, any observed association between attentiveness to fruit and vegetable intake and sleep efficiency should be interpreted as reflecting a possible link between health-oriented attentional engagement and sleep health, rather than as evidence that fruit and vegetable intake itself directly improves sleep efficiency. Despite these theoretical connections, empirical studies rarely examine whether self-reported attentional engagement in healthy dietary behavior is associated with objectively measured sleep outcomes, particularly when it is conceptualized as a behavioral marker rather than a direct measure of dietary intake.

The present cross-sectional study aimed to examine whether self-reported attentiveness to fruit and vegetable intake was associated with actigraphy-measured sleep efficiency among women aged 45 years and older. By incorporating actigraphy-based sleep assessment and adjusting for relevant sociodemographic and health-related covariates, this study extends existing literature beyond intake quantity and explores whether a simple behavioral marker of attention to healthy eating is related to sleep efficiency in midlife and older age. This framing allows the findings to be interpreted within a broader behavioral framework involving health consciousness, self-regulation, and lifestyle regularity while also acknowledging that causal and diet-specific interpretations remain limited. In doing so, the study seeks to contribute to the broader understanding of modifiable behavioral correlates of sleep health in women across the aging process.

## 2. Materials and Methods

### 2.1. Study Design and Ethical Approval

This cross-sectional study investigated dietary behaviors and objectively measured sleep parameters among women aged 45 years and older. The present analysis specifically examined whether self-reported behavioral attention to daily fruit and vegetable intake was independently associated with sleep efficiency.

Data were collected between 15 April and 4 June 2021. The study protocol was approved by the Institutional Review Board of National Cheng Kung University (IRB No.: B-ER-109-362). All participants provided written informed consent prior to participation.

### 2.2. Participants and Recruitment

Participants were recruited through community centers using convenience and snowball sampling methods. Eligible participants were women aged 45 years or older who were able to communicate in Mandarin or Taiwanese and independently complete questionnaires and sleep/activity logs. Exclusion criteria included institutional residence, inability to ambulate independently, severe sensory impairment, diagnosed dementia, current severe psychiatric illness, active cancer, or acute hospitalization during the study period. Participants were instructed to wear a wrist-worn actigraphy device continuously for seven consecutive days and to complete daily sleep logs. Follow-up contact was conducted on Day 3 to ensure adherence to device wear and diary completion. Devices and logs were collected on Day 7 via home visit or mail return. To ensure data quality, actigraphy data were considered valid if total wear time exceeded 7200 min and included at least five valid days of recording. Participants not meeting these criteria were excluded from analysis. The final analytical sample consisted of 143 participants.

### 2.3. Actigraphy Measurement and Sleep Efficiency

Sleep parameters were assessed using a wrist-worn actigraphy device developed by National Cheng Kung University (Tainan, Taiwan) research teams and equipped with an accelerometer to record movement data in 10 s epochs [24]. Raw actigraphy signals were processed using biomedical signal processing procedures and transformed into activity count data for subsequent statistical analysis, in accordance with validated methods for deriving actigraphy-compatible counts from raw acceleration signals [25]. The device has previously been validated against polysomnography (PSG), supporting its use for sleep–wake assessment and sleep parameter estimation in research settings [24].

Sleep efficiency (SE) was defined as the proportion of total sleep time (TST) to time in bed (TIB), expressed as a percentage and calculated as TST/TIB × 100%. Sleep diaries were used to verify bedtime and wake time and to identify non-wear periods during actigraphy monitoring.

### 2.4. Exposure Variable: Attention to Daily Fruit and Vegetable Intake

The primary exposure variable was a single-item, self-reported measure of attention to daily fruit and vegetable intake. Participants selected one of four response categories: (1) almost every day, (2) often, (3) occasionally, and (4) no such habit. This variable was intended to capture habitual attentiveness to, or routine monitoring of, daily fruit and vegetable intake in everyday life rather than actual intake quantity, dietary adequacy, or adherence to a quantified intake recommendation. Accordingly, it should be interpreted as a behavioral indicator of self-reported dietary attention rather than a direct measure of fruit and vegetable consumption. For regression analyses, “almost every day” was designated as the reference group.

### 2.5. Covariates

Covariates were selected based on theoretical relevance and prior literature and were entered hierarchically into regression models. Sociodemographic factors included age, education level, and employment status. Age was treated as a continuous variable. Education level was categorized as junior high school or below, high school/vocational school, and university/college or above. Employment status was categorized as currently employed or not currently employed.

Health-related and activity-related variables included body mass index (BMI), exercise habit, moderate-to-vigorous physical activity (MVPA), morning activity, and afternoon activity. BMI was calculated as weight in kilograms divided by height in meters squared and was treated as a continuous variable. Exercise habit was assessed by asking participants how many times they exercised per week and was categorized as regular exercise, occasional exercise, or no exercise. Regular exercise was defined as exercising 4–7 times per week, occasional exercise as exercising 1–3 times per week, and no exercise as 0 times per week. MVPA, morning activity, and afternoon activity were derived from actigraphy-based activity data. Morning activity was defined as mean activity counts between 05:00 and 12:00, and afternoon activity was defined as mean activity counts between 12:00 and 18:00.

Psychological factors included depressive symptoms and subjective physical age. Depressive symptoms were assessed using the 10-item short form of the Center for Epidemiologic Studies Depression Scale (CES-D) used in the Taiwan Longitudinal Study on Aging. Each item was scored from 0 to 3 according to symptom frequency during the past week, with positively worded items reverse-coded. Total scores ranged from 0 to 30, with higher scores indicating more depressive symptoms. Previous research has supported the psychometric properties and screening utility of this short-form CES-D among older adults in Taiwan [26]. Subjective physical age was assessed using a visual scale that asked participants to compare their perceived physical age with their actual age. Responses were categorized as younger than actual age, similar to actual age, or older than actual age.

All covariates were retained in the regression models based on conceptual importance rather than statistical significance.

### 2.6. Statistical Analysis

Descriptive statistics were calculated for all variables. Continuous variables are presented as means and standard deviations and categorical variables as frequencies and percentages. Group differences in participant characteristics by sleep efficiency status were examined using independent *t*-tests for continuous variables and chi-square tests for categorical variables.

Hierarchical multiple linear regression models were conducted to evaluate the association between self-reported attentiveness to fruit and vegetable intake and sleep efficiency. Covariates were sequentially entered according to conceptual domains, followed by the exposure variable in the final model. Categorical variables were dummy-coded before inclusion in the linear regression models, and reference categories were specified as shown in the regression table. Model fit was evaluated using the coefficient of determination (R^2^) and change in R^2^ (ΔR^2^) at each step. Standardized regression coefficients (β) and corresponding *p*-values were reported. To further consider sample size adequacy and model stability, we examined the ratio of the analytic sample size to the number of predictors in the fully adjusted model. The final model included 11 conceptual predictors and 16 dummy-coded predictor parameters, corresponding to approximately 8.9 participants per predictor parameter. This ratio was considered acceptable for exploratory linear regression analysis based on prior simulation evidence regarding the number of subjects per variable required in linear regression analyses [27]. Nevertheless, given the modest sample size, the regression findings were interpreted as exploratory rather than confirmatory.

To support model interpretation, assumptions of linear regression were evaluated before interpreting the final models. These included residual normality, linearity, homoscedasticity, and multicollinearity. Variance inflation factors (VIFs) were inspected to assess multicollinearity among predictors, and all VIF values were below 5.0, indicating no evidence of severe multicollinearity. All statistical analyses were performed using SPSS version 22.0. Statistical significance was defined as a two-sided *p*-value < 0.05.

## 3. Results

### 3.1. Participant Characteristics

The sample included 143 community-dwelling middle-aged and older women. The mean age was 56.34 ± 7.67 years, with a mean BMI of 23.62 ± 3.41 kg/m^2^. The average sleep efficiency was 82.69 ± 8.60%. More than half of the participants had a university or college education (55.2%), and 62.2% were currently employed. Regarding health behaviors, 30.1% reported regular exercise, and 29.4% reported often paying attention to daily fruit and vegetable intake. Detailed descriptive characteristics of the study population are presented in Table 1.

### 3.2. Sleep Efficiency Group Comparisons

Sleep efficiency was dichotomized into high and low groups using the sample median as the cutoff. Significant differences were observed between groups in age (*p* < 0.001), education level (χ^2^(2) = 6.47, *p* = 0.039), employment status (χ^2^(1) = 6.15, *p* = 0.013), self-rated health (*p* = 0.017), and attention to daily fruit and vegetable intake (χ^2^(3) = 17.13, *p* = 0.001). Detailed comparisons of participant characteristics by sleep efficiency group are presented in Table 2.

### 3.3. Hierarchical Multiple Linear Regression Predicting Sleep Efficiency

Hierarchical multiple linear regression analyses were conducted to examine the association between attention to daily fruit and vegetable intake and sleep efficiency (Table 3). Age was significantly and negatively associated with sleep efficiency (β = −0.30, *p* = 0.001). After adjustment for health behavior variables, BMI was inversely associated with sleep efficiency (β = −0.18, *p* = 0.032). Subjective physical age categorized as “older” remained positively associated with sleep efficiency (β = 0.28, *p* = 0.022).

**Figure 1:**
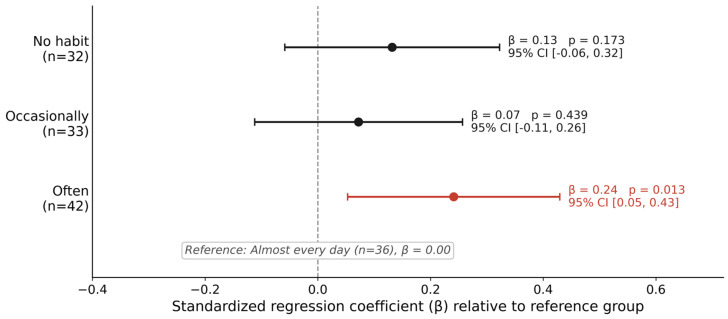
Adjusted standardized regression coefficients for self-reported attentiveness to daily fruit and vegetable intake in the final hierarchical regression model predicting sleep efficiency. The reference group was “almost every day.” Error bars represent 95% confidence intervals. The vertical dashed line indicates the null value (β = 0). Estimates should be interpreted as exploratory pairwise contrasts relative to the reference group, rather than evidence of a statistically significant trend across categories.

In the final model, participants who reported “often” paying attention to fruit and vegetable intake had significantly higher sleep efficiency compared with those who reported doing so “almost every day” (β = 0.24, *p* = 0.013). The final model explained 32% of the variance in sleep efficiency (R^2^ = 0.32). However, the incremental contribution of the dietary attentiveness block was marginal (ΔR^2^ = 0.04, *p* = 0.08), suggesting that the category-specific association should be interpreted cautiously. The category-specific regression estimates for dietary attentiveness are presented in Table 3; these estimates should be interpreted as exploratory pairwise contrasts rather than evidence of a statistically significant trend across categories. To facilitate interpretation of the dietary attentiveness coefficients in the final model, these category-specific estimates are additionally presented graphically in Figure 1.

## 4. Discussion

This cross-sectional study examined whether self-reported attention to daily fruit and vegetable intake was independently associated with objectively measured sleep efficiency among community-dwelling middle-aged and older women. Importantly, this exposure was not interpreted as a direct indicator of actual fruit and vegetable intake or dietary adequacy but as an exploratory behavioral marker of perceived attention to health-oriented eating. After adjustment for sociodemographic characteristics, health-related behaviors, and psychological factors, participants who reported “often” paying attention to fruit and vegetable intake had significantly higher sleep efficiency than those who reported doing so “almost every day.” In addition, older age was independently associated with lower sleep efficiency, which is consistent with previous evidence showing age-related changes in sleep structure and circadian regulation in midlife and later life [2,3]. Taken together, these findings suggest that the behavioral dimension of healthy eating may be associated with sleep efficiency beyond commonly considered covariates.

The most notable finding was the non-linear pattern observed across response categories. Rather than showing a gradient in which more frequent attention was associated with progressively better sleep efficiency, the “often” group demonstrated more favorable sleep efficiency than the “almost every day” reference group. This pattern should be interpreted cautiously. Because the interpretation of this contrast depends on the selected reference category, the finding should not be taken as evidence that the “often” group was superior to all other dietary attentiveness categories, including the “no habit” group, nor as evidence of a statistically significant trend across the four response categories. In addition, the overall incremental contribution of the dietary attentiveness block was marginal. It should not be interpreted as evidence of an optimal level of dietary attentiveness or a biologically meaningful threshold. Because the exposure captured self-reported attentiveness rather than quantified intake, it may reflect a sustainable level of dietary monitoring or routine adherence rather than maximal intake per se. It is also possible that the “almost every day” category was interpreted more heterogeneously across participants, thereby attenuating any linear gradient. For some participants, endorsement of “almost every day” may have reflected consistent dietary monitoring, whereas for others, it may have reflected heightened concern or disease-related dietary vigilance. Therefore, the observed pattern may partly reflect response interpretation variability or residual confounding rather than a definitive behavioral threshold. More broadly, attentional engagement in healthy eating may co-occur with self-regulatory capacity, routine stability, and other clustered health-promoting behaviors, all of which are plausibly relevant to sleep–wake regularity [21,22,23]. At the same time, these interpretations remain inferential, as the present study did not directly measure self-regulation, routine regularity, or health consciousness.

Although direct comparison should be made cautiously, the non-linear pattern observed in the present study is not entirely without precedent. In a prospective study of community-dwelling older adults in England, moderate fruit and vegetable consumption, rather than the highest level of intake, was associated with a lower risk of incident prefrailty and frailty, with the authors describing a U-shaped association [28]. That study examined actual intake quantity and frailty rather than dietary attentiveness and sleep efficiency and therefore does not directly explain our findings. Nevertheless, it provides a useful point of comparison by suggesting that fruit- and vegetable-related exposures may not always show simple linear associations with health outcomes in older adults. In this context, our findings may indicate that moderate, sustainable attentional engagement in healthy eating is more relevant to everyday behavioral regulation than endorsement of the most extreme self-reported category.

Our findings also align more broadly with prior literature linking fruit- and vegetable-related behaviors and overall diet quality to sleep outcomes. Previous studies have shown that healthier dietary patterns and higher diet quality are associated with better sleep quality and related sleep indicators [10,11,12,15,16]. In particular, Lee et al. [15] reported that older adults who consumed fruits and vegetables only occasionally had lower odds of good sleep quality than daily consumers, supporting the broader relevance of fruit- and vegetable-related behaviors to sleep health. Although that study did not examine dietary attentiveness or demonstrate the same non-linear contrast observed here, it is consistent with the view that fruit- and vegetable-related behaviors are meaningfully linked to sleep in later life. Several mechanisms may help explain these associations. Fruits and vegetables are rich in vitamins, minerals, and bioactive compounds, and prior studies suggest that some of these nutrients, including vitamin D, B vitamins, zinc, polyphenols, flavonoids, and coenzyme Q10, may be associated with improved sleep-related outcomes [29,30,31]. Although our measure did not assess actual intake amount, greater attention to daily fruit and vegetable intake may correspond, at least to some extent, to healthier dietary practices, but this possibility requires confirmation using validated dietary assessment tools.

At the same time, the direction of association cannot be assumed to run only from diet-related behavior to sleep. Prior evidence suggests that insufficient sleep may influence appetite regulation and food choice [32,33,34], whereas adequate sleep has been associated with healthier food choices [35]. Review-level and day-level evidence also suggest that sleep and fruit and vegetable consumption may be bidirectionally related [36,37]. Accordingly, individuals with better sleep efficiency may also be better able to maintain routine health-monitoring behaviors, including attention to dietary practices. Given the cross-sectional design of the present study, reverse directionality remains plausible.

This study has several strengths. Sleep efficiency was assessed objectively using a wrist-worn actigraphy device over multiple days, providing a more robust estimate of habitual sleep than single-time-point self-report measures. In addition, the analytic approach allowed the association between attention to fruit and vegetable intake and sleep efficiency to be examined while accounting for a range of conceptually relevant covariates. Several limitations should also be acknowledged. First, the cross-sectional design precludes causal inference and does not allow determination of temporal ordering. Second, the exposure was measured using a single self-report item and likely captured perceived attentiveness rather than actual intake quantity. As a result, the measure may also reflect related constructs such as health consciousness, routine self-monitoring, or general lifestyle organization, introducing potential construct ambiguity, misclassification, and reporting bias. Third, because detailed dietary intake data were unavailable, it was not possible to determine whether the observed association reflected actual dietary composition, attentional orientation toward healthy eating, or other correlated health behaviors. Fourth, residual confounding cannot be excluded, particularly from unmeasured factors such as routine regularity, chronic disease management, or psychosocial characteristics. In addition, several potentially important factors, including chronic medical conditions, medication use, menopausal status, caffeine intake, alcohol consumption, and sleep disorders, were not fully measured or controlled and may have influenced sleep efficiency. Fifth, the use of convenience and snowball sampling may have introduced selection bias and may limit the external validity of the findings. In addition, because “almost every day” served as the reference category, the apparent advantage of the “often” group may partly reflect differences in how participants interpreted the response options rather than a true behavioral threshold. Finally, the marginal *p*-value for the block-level model change (*p* = 0.08) suggests that the overall contribution of the attention variable warrants cautious interpretation, and individual group comparisons should be considered exploratory.

Future studies should use longitudinal designs, incorporate more detailed or objective dietary assessments, and include measures of self-regulation, health consciousness, and daily routine stability to clarify the mechanisms underlying this association. Replication in larger and more diverse samples will also be important, particularly to determine whether the observed advantage in the “often” group reflects a meaningful behavioral phenomenon or instability in how participants interpreted the response options. If replicated, a brief measure of dietary attentiveness may serve as a useful hypothesis-generating marker for identifying individuals with potentially different sleep-health profiles.

## 5. Conclusions

Self-reported attentiveness to daily fruit and vegetable intake showed a category-specific association with actigraphy-derived sleep efficiency after adjustment for relevant covariates among community-dwelling middle-aged and older women. The finding that the “often” group showed higher sleep efficiency than the “almost every day” group suggests a potentially non-linear association; however, this pattern should be interpreted cautiously because actual dietary intake was not measured. Longitudinal studies using more detailed dietary and behavioral measures are needed to clarify directionality and underlying mechanisms.

## Figures and Tables

**Table 1:** Participant characteristics (*N* = 143).

Variable	Total Sample
Age, years	56.34 ± 7.67
BMI, kg/m^2^	23.62 ± 3.41
Sleep efficiency, %	82.69 ± 8.60
Education level, *n* (%)	
Junior high school or below	16 (11.2)
High school/vocational	48 (33.6)
University/college or above	79 (55.2)
Employment status, *n* (%)	
Currently employed	89 (62.2)
Unemployed	54 (37.8)
Exercise habit, *n* (%)	
Regular	43 (30.1)
Occasional	79 (55.2)
None	21 (14.7)
Attention to daily fruit and vegetable intake, *n* (%)	
Almost every day	36 (25.2)
Often	42 (29.4)
Occasionally	33 (23.1)
No habit	32 (22.4)

Note. Continuous variables are presented as mean ± SD. Categorical variables are presented as *n* (%). Attention to daily fruit and vegetable intake was a self-reported behavioral measure, not a measure of actual intake or dietary adequacy.

**Table 2:** Comparison of participant characteristics by sleep efficiency group (*N* = 143).

Variable	High SE (*n* = 72)	Low SE (*n* = 71)	Test Statistic	*p*-Value
Age (years)	53.88 ± 6.40	58.83 ± 8.07	t(141) = −4.07	<0.001
Education level			χ^2^(2) = 6.47	0.039
Junior high or below	4 (5.6%)	12 (16.9%)		
High school/vocational	22 (30.6%)	26 (36.6%)		
University/college	46 (63.9%)	33 (46.5%)		
Employment status			χ^2^(1) = 6.15	0.013
Currently employed	52 (72.2%)	37 (52.1%)		
Unemployed	20 (27.8%)	34 (47.9%)		
Self-rated health			χ^2^(1) = 5.73	0.017
Fair/poor	40 (55.6%)	53 (74.6%)		
Good/excellent	32 (44.4%)	18 (25.4%)		
Attention to fruit and vegetable intake			χ^2^(3) = 17.13	0.001
No habit	15 (20.8%)	17 (23.9%)		
Occasionally	13 (18.1%)	20 (28.2%)		
Often	32 (44.4%)	10 (14.1%)		
Almost every day	12 (16.7%)	24 (33.8%)		

Note. Continuous variables were compared using independent *t*-tests; categorical variables were compared using chi-square tests. Statistical significance was defined as *p* < 0.05. Attention to daily fruit and vegetable intake was a self-reported behavioral measure, not a measure of actual intake or dietary adequacy.

**Table 3:** Hierarchical multiple linear regression predicting sleep efficiency (*N* = 143).

Variable	Model 1		Model 2		Model 3		Model 4	
	β	*p*	β	*p*	β	*p*	β	*p*
Age	−0.30	0.001	−0.28	0.004	−0.30	0.002	−0.31	0.001
Education (ref: University/college)								
High school/vocational	−0.02	0.837	−0.03	0.738	−0.06	0.471	−0.04	0.619
Junior high or below	−0.22	0.010	−0.16	0.088	−0.16	0.074	−0.13	0.144
Employment (ref: Employed)								
Unemployed	0.01	0.888	−0.04	0.681	−0.01	0.908	0.00	0.995
BMI			−0.18	0.032	−0.14	0.118	−0.14	0.113
Exercise (ref: Regular)								
Occasional			0.01	0.922	0.03	0.709	0.05	0.577
None			−0.05	0.628	0.00	0.970	0.00	0.988
Moderate-to-vigorous activity			0.30	0.057	0.24	0.111	0.22	0.149
Morning activity			−0.16	0.186	−0.14	0.223	−0.09	0.428
Afternoon activity			−0.19	0.127	−0.19	0.130	−0.18	0.132
Depressive symptoms (CES-D)					−0.12	0.160	−0.09	0.270
Subjective physical age (ref: Younger)								
Similar					0.20	0.072	0.20	0.069
Older					0.28	0.022	0.28	0.019
Attention to fruit and vegetable intake (ref: Almost every day)								
Often							0.24	0.013
Occasionally							0.07	0.439
No habit							0.13	0.173
R^2^	0.17		0.21		0.28		0.32	
ΔR^2^			0.05		0.06		0.04	
*p* for model change			0.285		0.011		0.08	

Models were constructed sequentially according to conceptual domains. Categorical predictors were dummy-coded, with reference categories indicated in parentheses. β = standardized regression coefficient. Ref. = reference category. Statistical significance was defined as *p* < 0.05. Note. Attention to daily fruit and vegetable intake was a self-reported behavioral measure, not a measure of actual intake or dietary adequacy. For this variable, regression estimates represent dummy-coded contrasts relative to the “almost every day” reference group.

## Data Availability

The datasets generated and/or analyzed during the current study are not publicly available due to ethical and privacy considerations but are available from the corresponding author upon reasonable request.

## Abbreviations

The following abbreviations are used in this manuscript:

BMI	Body mass index
CES-D	Center for Epidemiologic Studies Depression Scale
MVPA	Moderate-to-vigorous physical activity
PSG	Polysomnography
SE	Sleep efficiency
TIB	Time in bed
TST	Total sleep time
